# Genome-wide identification and evolutionary analysis of leucine-rich repeat receptor-like protein kinase genes in soybean

**DOI:** 10.1186/s12870-016-0744-1

**Published:** 2016-03-02

**Authors:** Fulai Zhou, Yong Guo, Li-Juan Qiu

**Affiliations:** The National Key Facility for Crop Gene Resources and Genetic Improvement (NFCRI) and MOA Key Labs of Crop Germplasm and Soybean Biology (Beijing), Institute of Crop Science, Chinese Academy of Agricultural Sciences, No.12 Zhongguancun South Street, Haidian District Beijing, 100081 P. R. China

**Keywords:** Soybean, Leucine-rich repeat receptor-like kinase (LRR-RLK), Phylogenetic analysis, Expression profiling, Evolutionary analysis

## Abstract

**Background:**

Leucine-rich repeat receptor-like kinases (LRR-RLKs) constitute the largest subfamily of receptor-like kinases in plant. A number of reports have demonstrated that plant *LRR-RLKs* play important roles in growth, development, differentiation, and stress responses. However, no comprehensive analysis of this gene family has been carried out in legume species.

**Results:**

Based on the principles of sequence similarity and domain conservation, a total of 467 *LRR-RLK* genes were identified in soybean genome. The *GmLRR-RLKs* are non-randomly distributed across all 20 chromosomes of soybean and about 73.3 % of them are located in segmental duplicated regions. The analysis of synonymous substitutions for putative paralogous gene pairs indicated that most of these gene pairs resulted from segmental duplications in soybean genome. Furthermore, the exon/intron organization, motif composition and arrangements were considerably conserved among members of the same groups or subgroups in the constructed phylogenetic tree. The close phylogenetic relationship between soybean *LRR-RLK* genes with identified *Arabidopsis* genes in the same group also provided insight into their putative functions. Expression profiling analysis of *GmLRR-RLKs* suggested that they appeared to be differentially expressed among different tissues and some of duplicated genes exhibited divergent expression patterns. In addition, artificial selected *GmLRR-RLKs* were also identified by comparing the SNPs between wild and cultivated soybeans and 17 genes were detected in regions previously reported to contain domestication-related QTLs.

**Conclusions:**

Comprehensive and evolutionary analysis of soybean *LRR-RLK* gene family was performed at whole genome level. The data provides valuable tools in future efforts to identify functional divergence of this gene family and gene diversity among different genotypes in legume species.

**Electronic supplementary material:**

The online version of this article (doi:10.1186/s12870-016-0744-1) contains supplementary material, which is available to authorized users.

## Background

Receptor-like kinases (RLKs) are a diverse group of transmembrane proteins characterized with a ligand-binding domain to receive signal molecules, a membrane-spanning domain to anchor the protein, and a cytoplasmic protein kinase domain to transduce signals downstream [1]. In both plants and animals, RLKs mediate plenty of signaling messages at the cell surface and act as key regulators during developmental processes [2–4]. The first RLK of higher plant was isolated from maize and subsequently numerous RLKs have been identified from more than 20 plant species [5]. In plant, the superfamily of RLKs is divided into three major groups based on the presence or absence of the receptor and kinase domain [1, 6, 7]. According to the divergence of extracellular domains, RLKs can be further classified into 17 subgroups, including leucine-rich repeat (LRR) RLKs, S-domain RLKs, and so on [8, 9]. Among all these subgroups, LRR-RLK is the largest one in plants by far, the members of which contain several tandem repeats of about 24 amino acids with conserved leucine residues in the extracellular regions [7, 10].

Genetic and biochemical studies have demonstrated that plant *LRR-RLKs* play important roles in diverse processes during growth and development [11, 12]. In *Arabidopsis*, *LRR-RLKs* including *SERK1*/*2, EMS1*, *BAM1*/*2*, *RPK2* and *FER* have been proved to modulate the processes of anther development and fertilization [13–18]. Enough evidences supported that CLV and RPK2 were essential receptor-like kinases in formation and maintenance of shoot apical meristem [19, 20]. Some other reports also revealed that *LRR-RLK* genes such as *BRI* and *BAK1* were involved in brassinosteroid signaling transduction while a few other *LRR-RLK* genes were associated with the stress responses of abscisic acid [21–23]. Moreover, some *LRR-RLK* genes were also reported to possess dual functions due to the cross talks between plant development and defense processes or the recognition of multiple ligands by one receptor [2]. For example, *Arabidopsis ERECTA* gene has been characterized not only to regulate ovule development [24] but also to be involved in resistance to bacterial wilt [25].

The rapidly increasing sequenced genomes have facilitated identification of whole gene family by bioinformatics tools at genomic level in plant. To date, the structure features and expression profiles of *LRR-RLK* genes have been described in plants including *Arabidopsis* [26], rice [27], and poplar [28]. In most of these species, LRR-RLKs appeared to be large families with hundreds of members and evolved to perform diverse functions [28–30]. Some reports also revealed that *LRR-RLK* genes had redundant functions due to extensive gene duplication in genome. For example, although single mutant of *serk1* or *serk2* displays normal anther morphology, *serk1 serk2* double mutant could rescue the phenotype of *exs* or *ems* mutants which failed to form pollen due to the absence of tapetal cell layer and production of extra sporogenous cells in *Arabidopsis* [13, 14]. Translational fusion study of SERK1/SERK2 to variants of green fluorescent protein also suggested that SERK1/SERK2 may function as part of a protein complex [13].

Soybean (*Glycine max*) is the most important legume source of protein for animal feed and economic source of vegetable oil for human nutrition [31]. During the evolutionary history, soybean genome underwent two rounds of whole genome duplication (WGD) approximately 59 and 13 million years ago (MYA) [32]. Unlike most of other diploids, nearly 75 % of genes exhibit multiple copies in soybean genome due to the lack of immediate diploidization during the relatively recent WGD [33]. Therefore, the structure features of most gene families in soybean are more complex than in *Arabidopsis*, rice or poplar. Although only a few members of *LRR-RLK* genes have been functionally characterized in soybean, enough evidences supported that soybean *LRR-RLK* genes also played important roles in various plant development and defense processes including leaf senescence, cell elongation, and cold stress tolerance [34–36].

In the present study, a genome-wide search for *LRR-RLK* genes was performed in soybean and a total of 467 *GmLRR-RLKs* had been identified. Detailed analysis of genome organization, sequence phylogeny, gene structure, conserved domains, duplication status, and expression profiling were carried out. In addition, the evolutionary patterns of the *LRR-RLK* gene family were examined in soybean by analysis of genes in tandem and segmental duplication regions. Moreover, the effect of artificial selection in soybean *LRR-RLK* gene family was also detected during soybean domestication. Our results provide a framework for further evolutionary and functional characterization of the *LRR-RLK* gene family in soybean.

## Results and discussions

### Identification and genome distribution of *LRR-RLK* gene family in soybean

In order to identify all members of LRR-RLKs in soybean genome, a batch BLAST search was performed against soybean protein database using the amino acid sequences of all *Arabidopsis* LRR-RLKs as queries. All of the retrieved soybean proteins were then submitted to SMART and PFAM databases for annotation of the domain structure. Only candidate containing at least one LRR domain and a kinase domain was regarded as a “true” LRR-RLK. After removing of the unsupported sequences and redundant genes manually, a total of 467 putative *LRR-RLK* genes were identified from the whole genome of soybean. The identified soybean *LRR-RLK* genes encode peptides ranging from 423 to 1563 a.a. in length. Detailed information for each gene, including the accession number and the characteristics of the encoded protein, was listed in Additional file 1. Among all these putative GmLRR-RLKs, only three proteins (Glyma.03G026800, Glyma.07G047200 and Glyma.13G228300) were predicted to have two kinase domains. Comparing with *LRR-RLK* genes identified in *Arabidopsis*, rice and *populus* genome (213, 309 and 379 members respectively) [26–28], soybean *LRR-RLK* gene family identified in this study is the largest one in plant so far. The number of *GmLRR-RLKs* is about 2.2 fold of that of *AtLRR-RLKs*, which is consistent with the ratio of putative soybean homologs to each *Arabidopsis* gene [32, 37].

**Fig. 1:**
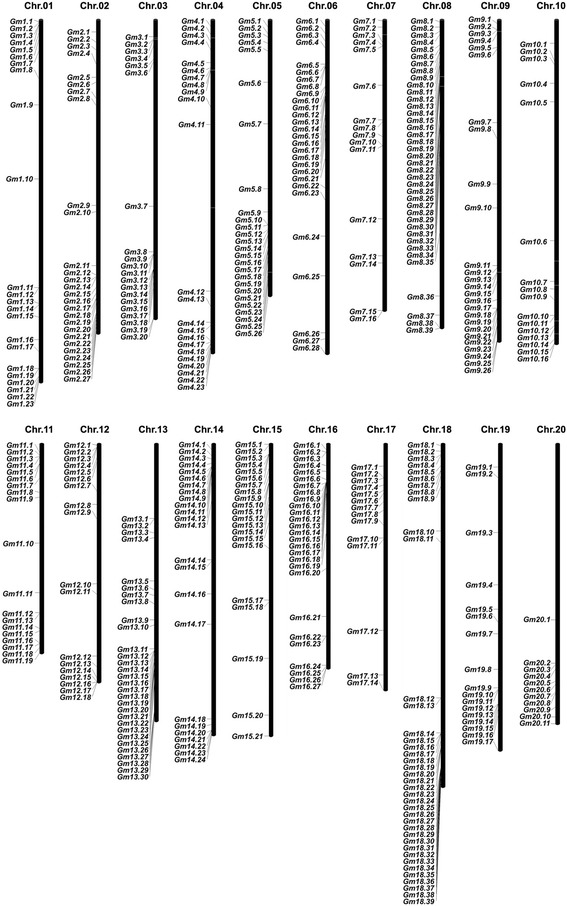
Genomic distribution of *LRR-RLK* genes across soybean chromosomes. Chromosomal locations of *GmLRR-RLKs* were indicated based on the physical position of each gene. The positions of genes on each chromosome were drawn with MapInspect software and the number of chromosome was labeled on the top of each chromosome

Physical positions of *GmLRR-RLKs* obtained from the Phytozome database (Additional file 1) were used to map them onto corresponding chromosomes of soybean. Results showed that 464 out of all soybean *LRR-RLK* genes could be mapped on all chromosomes from chromosome 1 to 20 (Fig. 1) while three other genes could be only mapped to unassembled genomic sequence scaffolds. Although every chromosome contained a certain number of *LRR-RLK* genes, the distribution of them appeared to be uneven across different chromosomes. The distribution ratio for each chromosome ranged from 2.4 % (11 members on chromosome 20) to 8.4 % (39 members on chromosomes 8 and 18). This distribution pattern is similar with other gene families in soybean and *LRR-LRK* gene families in other plant species [26–28, 38, 39].

### Phylogenetic analysis of soybean LRR-RLKs

**Fig. 2:**
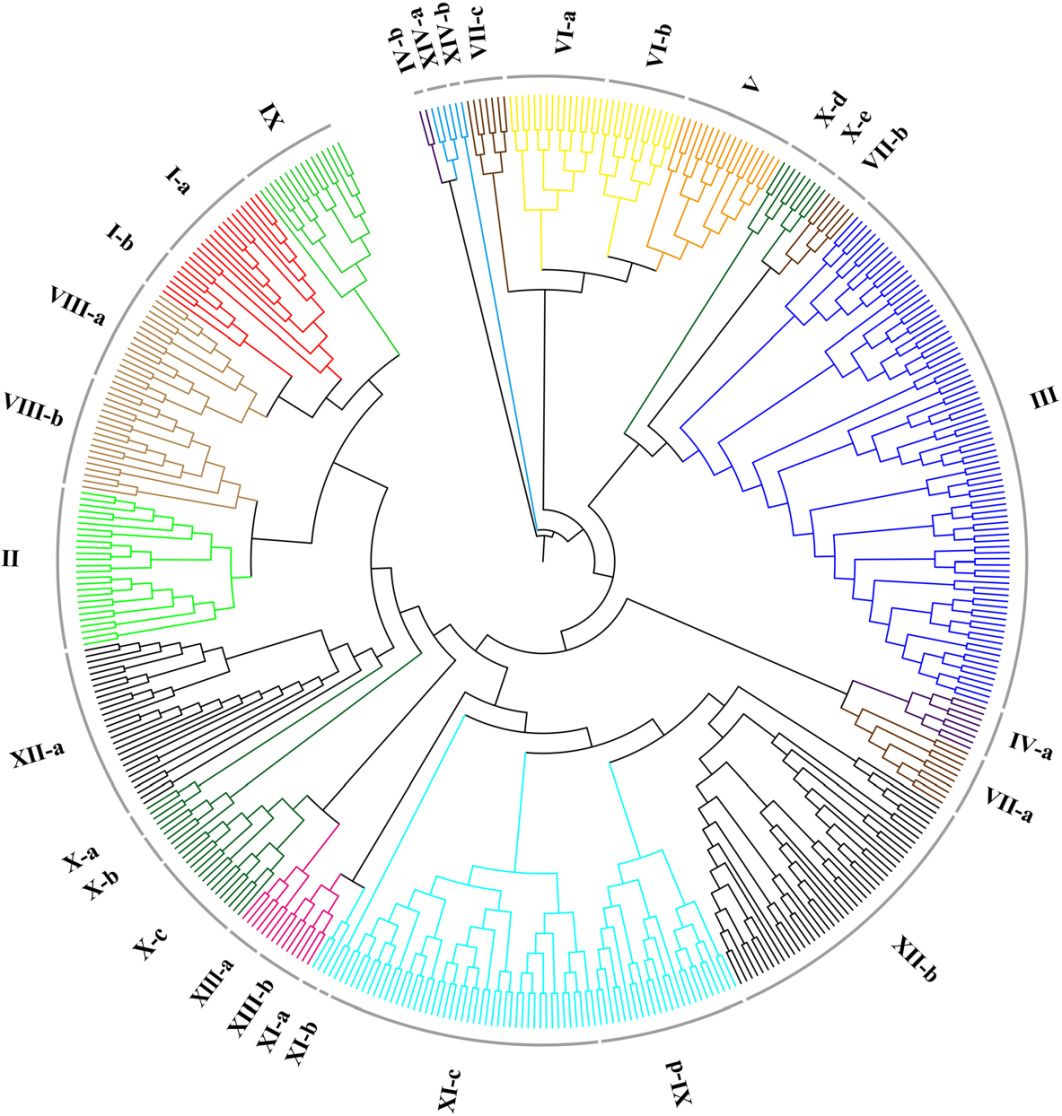
Phylogenetic analysis of LRR-RLKs retrieved from soybean. The amino acid sequences of kinase domains for 467 GmLRR-RLKs were aligned by Clustal X 1.8.3 and the phylogenetic tree was constructed using MEGA 6.0 by the neighbor-joining method with 1000 bootstrap replicates. All soybean LRR-RLKs were classified into 14 distinct groups based on the nomenclature of *Arabidopsis* LRR-RLKs (from I to XIV)

To study the evolutionary relationships of LRR-RLK members in soybean, the amino acid sequences of kinase domains from all GmLRR-RLKs were used to perform a multiple alignment with Cluster X and a phylogenetic tree was constructed using MEGA (Fig. 2). The phylogenetic tree showed that all GmLRR-RLKs could be classified into different groups or subgroups according to the nodes of the tree. When all the GmLRR-RLKs were clustered with all AtLRR-RLKs (Additional file 2), the members of each soybean LRR-RLKs group were determined according to the nomenclature of the *Arabidopsis* homologues within the same group (Table 1 and Fig. 2). Interestingly, some members of GmLRR-RLKs exhibited soybean specific features due to high level of duplication in genome. For examples, although only two members of *Arabidopsis* LRR-RLKs (AT1G35710 and AT4G08850) in the subgroup XII-b, as many as 45 *GmLRR-RLKs* were identified as the orthologous genes of these two *AtLRR-RLKs* (Additional file 2). The rapid expansion of GmLRR-RLKs in subgroup XII-b may result from two large gene clusters in Chromosomes 16 and 18.

**Table 1:** The classification of groups and subgroups for soybean LRR-RLK proteins

Groups	Subgroups	No. of Genes	Length of amino-acid (a.a.)	Percentage with signal peptide
I	a	17	427–905	82.4 %
	b	6	919–950	66.7 %
II		26	568–644	96.2 %
III		88	589–1065	76.1 %
IV	a	8	677–689	100.0 %
	b	2	982–984	100.0 %
V		18	633–802	83.3 %
VI	a	16	628–720	68.8 %
	b	13	634–838	69.2 %
VII	a	10	884–1007	70.0 %
	b	6	1109–1133	100.0 %
	c	7	665–856	85.7 %
VIII	a	17	879–987	82.4 %
	b	18	813–1036	88.9 %
IX		16	893–1355	75.0 %
X	a	4	1089–1155	50.0 %
	b	5	1042–1140	80.0 %
	c	15	990–1269	80.0 %
	d	5	423–631	60.0 %
	e	4	887–950	100.0 %
XI	a	3	1082–1086	100.0 %
	b	2	885–888	100.0 %
	c	44	631–1275	70.5 %
	d	24	443–1032	87.5 %
XII	a	28	443–1214	53.6 %
	b	45	545–1563	64.4 %
XIII	a	6	580–649	100.0 %
	b	8	980–1009	75.0 %
XIV	a	4	854–900	100.0 %
	b	2	955–960	50.0 %

Since most of the *AtLRR-RLKs* with similar functions have a tendency to cluster together, the soybean *LRR-RLK* genes in the same group or subgroup may have similar functions with their *Arabidopsis* homologs. Except for groups IV and VIII having no *Arabidopsis* ortholog with identified function, all the other groups have at least one *AtLRR-RLK* functional characterized. For example, *GmLRR-RLKs* in groups I, II, III, VII, and XII were clustered with *AtLRR-RLKs* involved in organ/tissue development and defense signaling [13, 14, 40–43]. Group V included the *Arabidopsis SCM* gene related to root hair specification and the *SRF* gene in cell wall biology [44, 45]. In addition, the *Arabidopsis LRR-RLK* genes involved in brassinosteroid and peptide signaling fell into the group X [46] and genes related to cell fate specification, organ morphogenesis [47], vascular development [48, 49], abscisic acid signaling, and defense response [50] were grouped in group XI. Moreover, subgroup XIII-a contained two *FEI* genes which were involved in signaling pathway of cell wall development [51], while subgroup XIII-b included *ERECTA* and *ERECTA-LIKE* genes regulating the stomata development and organ size [52].

### Gene structure and conserved motif analysis

**Fig. 3:**
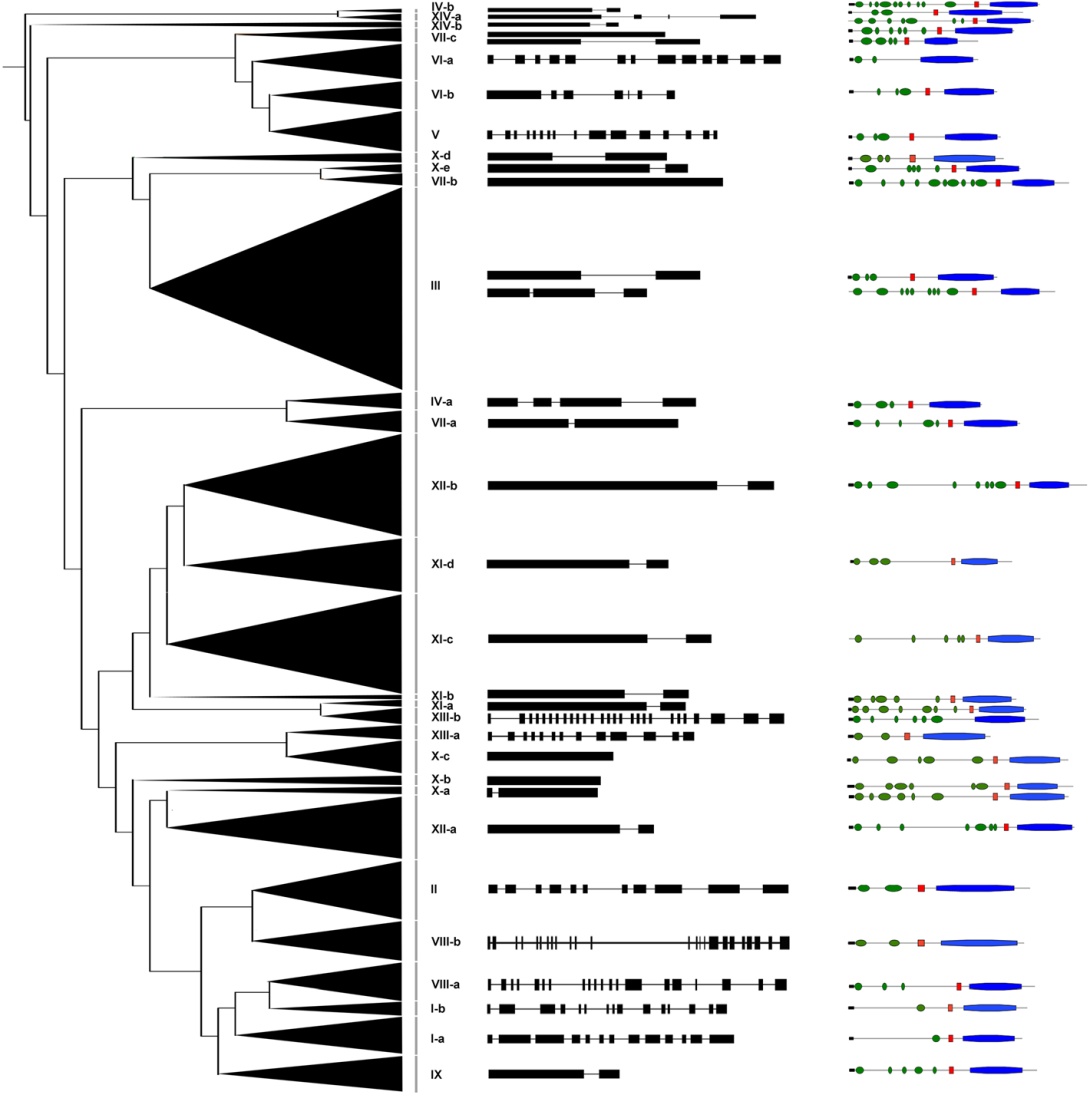
Representative exon/intron and motif structure of each LRR-RLK subgroup in soybean. Exons and introns are represented by black boxes and lines respectively. Signal peptide, transmembrane domain, and kinase domain are represented by black, red and blue boxes respectively. LRR motifs are indicated using green oval shapes. The relative size of each element can be estimated by the length of box or line

Since exon/intron diversification of members in a gene family always plays an important role in the evolution of this gene family [53], the exon/intron organization of individual soybean *LRR-RLK* gene was also analyzed. The results showed that nearly half members of *GmLRR-RLKs* (217 out of 467) had only one intron while 26 genes had only one exon. Two, three, four, and five introns were found in 46, 19, 4, and 4 soybean *LRR-RLK* genes. Meanwhile, a total of 151 genes had more than five introns and 96 out of them had more than ten (Additional files 1 and 3). In terms of intron number and length, most of *GmLRR-RLKs* in the same groups or subgroups have very conserved exon/intron organizations (Fig. 3). For instance, majority of soybean *LRR-RLK* genes in groups VII, X, and XI contain zero, one, and two introns except for only three members with four introns. However, the members of groups V, VI and XII displayed a large variability in either number or distribution of introns. Most interestingly, the members of subgroup XIII-b contain as many as 26 introns, which is about twice as many as that in the members of subgroup XIII-a. The exon/intron organization indicated the conservation within subgroup and divergence among different subgroups.

To further understand the potential functions of the *LRR-RLK* genes in soybean, all putative motifs of these proteins were predicted by using the program MEME (Multiple Em for Motif Elicitation). The results suggested that the motif compositions among groups or subgroups were consistent with the phylogenetic classification. Differences among groups or subgroups were observed in not only types of motifs but also number of specific motif in one protein (Additional file 4). In addition, searching for the possible signal peptides in all soybean LRR-RLKs using SignalP showed that 359 members have signal peptides. Meanwhile, the transmembrane (TM) domain was also predicted with TMHMM and a total of 442 GmLRR-RLKs had at least one while 25 members had no TM domain, among which 205 proteins had at least two TM domains. These results also indicated that most of the closely related members in the phylogenetic tree exhibited similar motif, which further suggested that a great deal of functional redundancy existed among soybean LRR-RLK proteins in the same subgroup (Fig. 3 and Additional file 5).

### Gene duplication and orthologous relationships of soybean *LRR-RLK* genes

Gene duplication is always considered to be one of primary driving forces during the evolution of genomes [54]. Segmental duplication, tandem duplication and transposition events are regarded as three main causes for the expansion of gene family in plant [55]. In our analysis, the tandem duplication cluster was defined as a region containing two or more soybean *LRR-RLK* genes within 200 kb. The results showed that about 20.3 % (94 out of 464) genes in this gene family were located in regions with tandem duplications and composed 33 clusters in total (Additional file 6). The largest tandem duplication cluster contained as many as ten genes while the smallest one contained only two. Further analysis also revealed that the tandem duplication clusters were distributed unevenly among 14 phylogenetic groups. Group XII contained the most clusters with eight clusters including 35 genes while Groups III, IV, V, VI, VII, IX, XIV had no cluster.

**Fig. 4:**
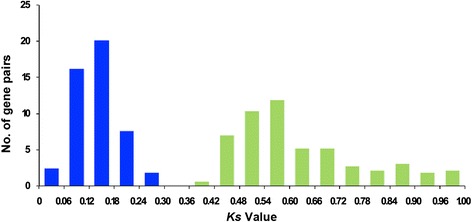
The distribution of *Ks* values in all segmental duplicated *GmLRR-RLKs*. The *Ks* value of each duplicated gene pair was calculated by using PGDD database (http://chibba.agtec.uga.edu/duplication/). The two peaks at 0.12-0.18 and 0.54-0.6 were consistent with whole genome duplication events of soybean at around 13 and 59 MYA

Segmental duplications generate duplicated genes through polyploidy followed by chromosome rearrangements [56]. Our results showed that a total of 329 putative paralogous gene pairs (340 genes or 73.3 % of total genes) were resulted from segmental duplications (Additional file 7), suggesting that segmental duplication might be the main mechanism of gene expansion in soybean LRR-RLK gene family. In order to estimate the date of the segmental duplication event, *Ks* value was used for calculating the separation time of each putative paralogous gene pair (Additional file 7). The distribution analysis of *Ks* values suggested that all the *Ks* values ranged from 0 to 1.0 with two peaks at 0.12–0.18 and 0.54–0.6 (Fig. 4). According to the clock-like rate of synonymous substitution in soybean, the segmental duplications of the soybean *LRR-RLK* genes originated from 0 to 81.8 MYA and the two peaks were consistent with whole genome duplication events at around 13 and 59 MYA [32]. In addition, the *Ka/Ks* ratios of 239 paralogous gene pairs were less than 0.3 while the other 90 gene pairs were all larger than 0.3, which demonstrated a possibility of significant functional divergence of some soybean *LRR-RLK* genes after the duplication events.

### Expression profiles of *LRR-RLK* genes in soybean

To gain a broader understanding of the putative functions of soybean *LRR-RLKs*, the expression profiles of these genes were examined by using the RNA-Seq dataset from different soybean tissues. The distinct transcript abundance patterns of all 467 LRR-RLK genes were identified from RNA-Seq atlas data of tissues including roots, root hairs, nodules, leaves, stems, flowers, SAM, pods, and seeds. Although some genes exhibited low transcript abundance like genes encoding transcription factors, most of them demonstrated distinct tissue specific expression pattern (Additional file 8). Detailed analysis showed that 53 (11.3 %), 68 (14.6 %), 65 (13.9 %), 53 (11.3 %), 95 (20.3 %), 87 (18.6 %), 75(16.1 %), 67 (14.3 %), and 51 (10.9 %) *GmLRR-RLKs* had specific transcript accumulation in roots, root hairs, nodules, leaves, stems, SAM, pods, seeds, and flowers respectively, suggesting that these *LRR-RLK* genes might function as tissue-specific regulators in different cells or organs.

**Fig. 5:**
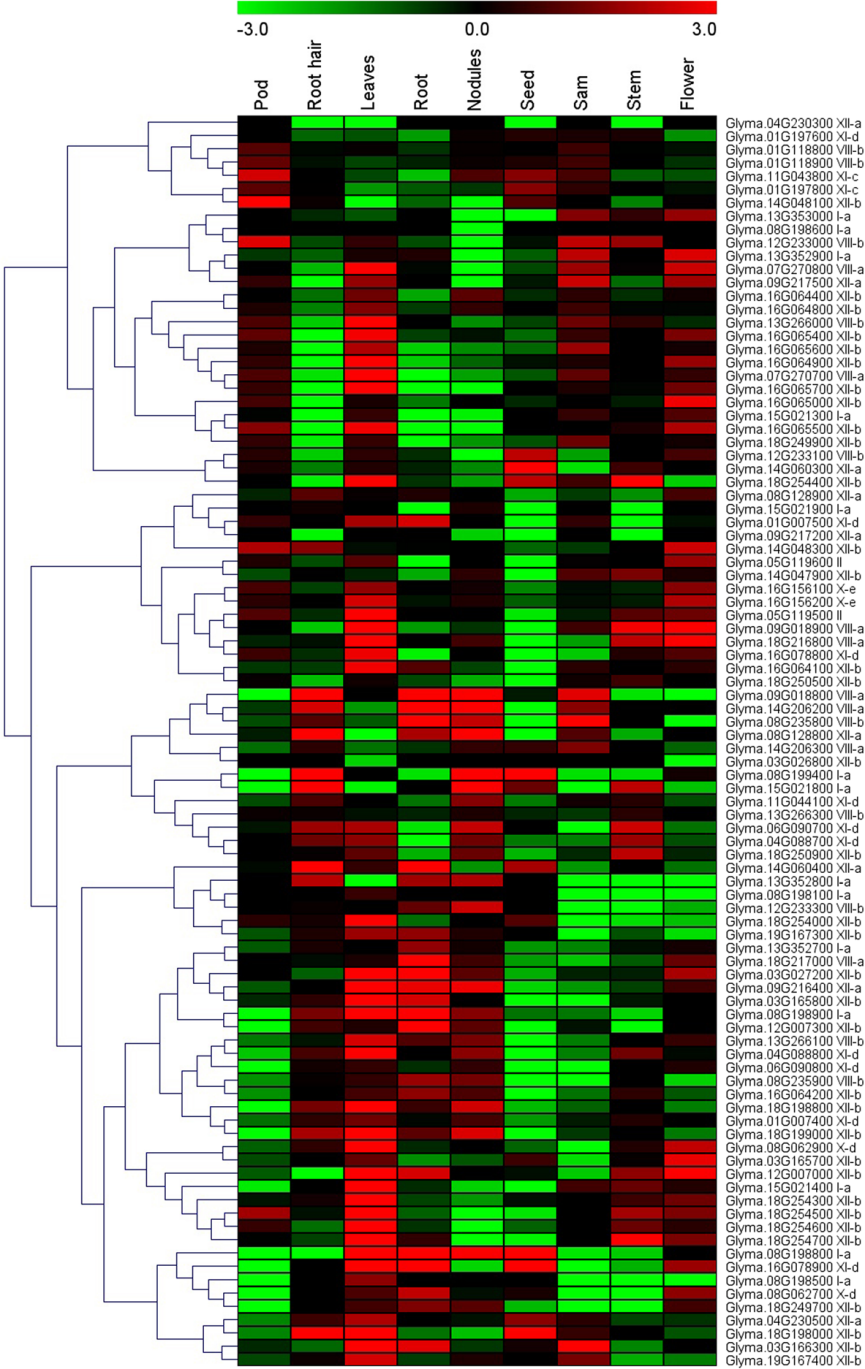
Expression pattern of *LRR-RLK* genes located in tandem duplication clusters. The RNA-seq data of each gene in pod, root hair, leaves, root, nodules, seed, stem, SAM, flower was gene-wise normalized and hierarchically clustered. The color scale above represents expression values, green indicating low levels while red indicating high levels of transcript abundance

Detailed analysis of the expression profiles also suggested that some *GmLRR-RLKs* clustered in the same subgroup had similar expression pattern. For example, all the *LRR-RLK* genes in subgroup XIII-b were mainly expressed in seeds and SAM, also indicating the existence of redundancy among the soybean *LRR-RLK* genes in these subgroups. However, it has also been reported that more than 50 % of duplicated *LRR-RLKs* exhibited expressional divergence in both rice and *Arabidopsis* [57, 58]. Our results showed that only 7 out of 33 clusters of tandem duplicated genes exhibited similar expression patterns in soybean (Fig. 5). In order to validate the expression patterns of these duplicated genes, the expression levels of randomly selected gene pairs were detected by using qRT-PCR. The result showed that similar or distinct expression patterns of these gene pairs identified by RNA-seq dataset were consistent with the results of qRT-PCR (Additional file 9). Moreover, among 329 pairs of LRR-RLK paralogs, only 50 pairs exhibited similar expression patterns and were likely to functionally substitute for each other.

### Artificial selection analysis for *LRR-RLKs* during soybean domestication

**Fig. 6:**
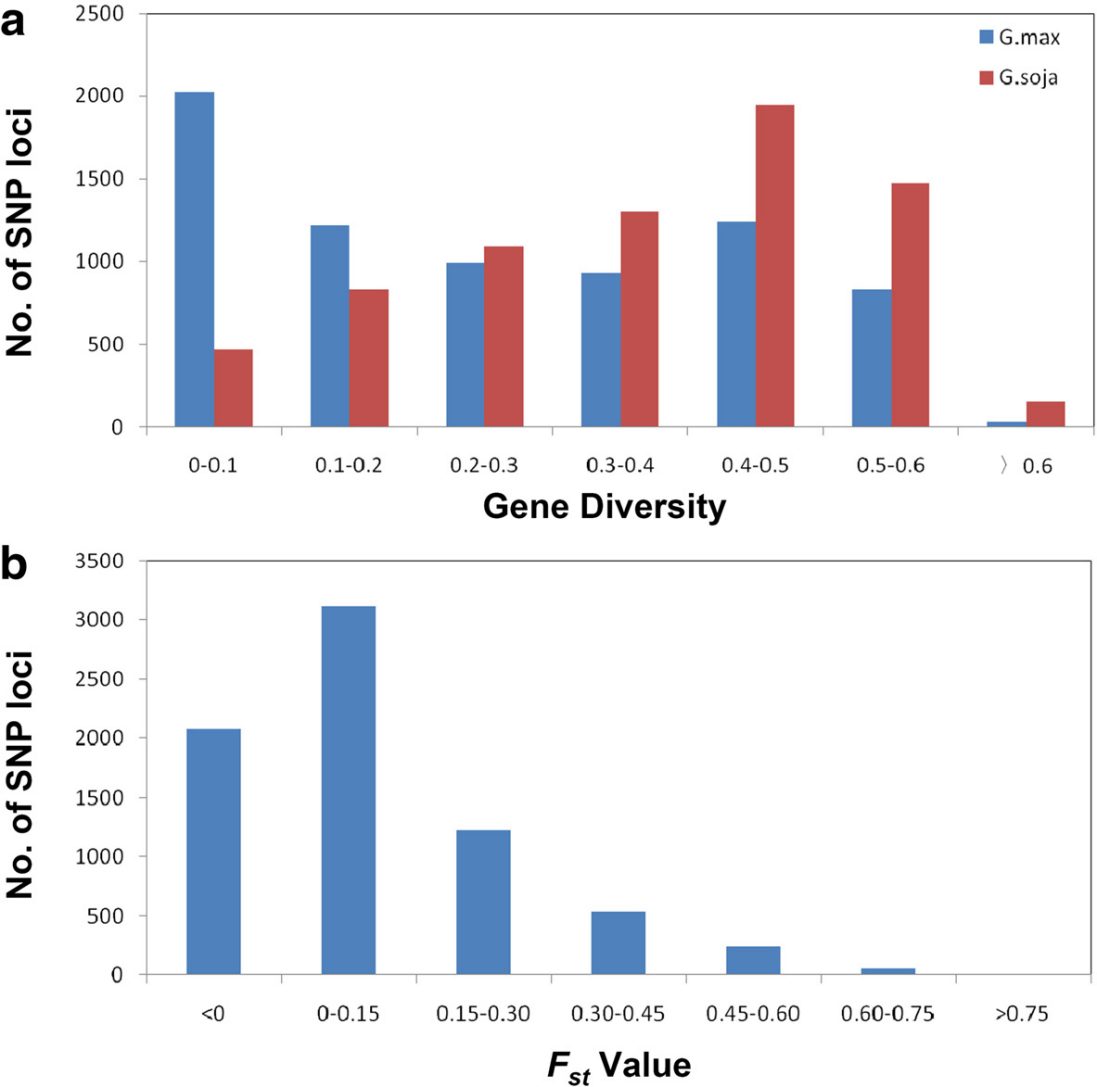
The distribution of gene diversities (**a**) and *F*_*st*_ values (**b**) for SNP loci located in all *GmLRR-RLKs*. The gene diversity and *F*_*st*_ value of each SNP were calculated by using Genepop V4.0. The gene diversities of most SNPs in *G.max* were less than 0.2 while most of them in *G.soja* were more than 0.4. SNPs with *F*_*st*_ value higher than 0.45 were regarded as selected loci

In order to analyze the selection effects of *GmLRR-RLKs* during soybean domestication, resequencing data of wild and cultivated soybeans were used [59, 60]. A total of 7239 SNPs have been identified in the genic regions of 407 soybean *LRR-RLK* genes based on the sequence diversity analysis between 35 cultivated soybeans (*G.max*) and 21 wild soybeans (*G.soja*) (Additional file 10). At these loci, the gene diversity was estimated at ~0.25 on average in cultivated population, which was significantly lower than that in wild population (~0.36). SNP149 in *Glyma.01G197800* is a typical example which has no diversity in cultivated soybeans while has diversity as high as 0.66 in wild soybeans. The distribution analysis also revealed that the gene diversities of most loci were less than 0.2 in *G.max* while 0.4–0.6 in *G.soja* (Fig. 6a), indicating that the gene diversities of these *LRR-LRKs* in cultivated soybean were reduced when compared with their wild progenitors.

In order to identify the selective *GmLRR-RLKs* during soybean domestication, *F*_*st*_ value of each locus was calculated between two populations (Fig. 6b and Additional file 10). The results showed that 71.6 % loci (5182 out of 7239 loci) underwent non-selection with *F*_*st*_ <0.15 during soybean evolution. However, a total of 302 SNPs in 98 soybean *LRR-RLK* genes were identified as selected loci with *F*_*st*_ value cutoff 0.45 (Additional file 11). Although a number of these SNPs (134 out of 302) were located in the introns of *GmLRR-RLKs*, nearly one third (89 SNPs) of them resulted in non-synonymous alteration. Further analysis showed that all subgroups of *GmLRR-RLKs* had selected genes except for group XIV. Group XI has the most selected soybean *LRR-RLK* genes (21 genes) while group IV has only one gene. Especially, although *Glyma.11G214400* and *Glyma.18G050700* have the largest number of selected SNPs (36 and 32 SNPs respectively), majority SNPs in the first gene resulted in non-synonymous alteration while most of SNPs appeared in the introns of the second one. Furthermore, a number of selected *LRR-RLK* genes between the wild and cultivated populations were detected in regions previously reported to contain domestication-related QTLs (Additional file 11). These included six *GmLRR-RLKs* located at QTLs related to pod traits including pod dehiscence/number/maturity [61, 62], five genes located at QTLs conditioning twinning habit [63–65], four genes at QTL regions of seed weight/hard-seededness and two genes at regions related to lodging [63, 65]. These selected genes reflected the important roles of *GmLRR-RLKs* on soybean domestication and contribute to the cultivation of soybeans in order to meet the demands of human beings.

## Conclusions

Here we performed comprehensive and evolutionary analyses of *LRR-RLK* gene family in soybean, and provided detailed information on its members. A total of 467 putative *LRR-RLK* genes were identified in the soybean genome, which represented the largest *LRR-RLK* gene family identified in plant so far and a relatively large gene family in soybean. The distribution of all these genes was non-random across all soybean chromosomes and majority of them were located in segmental duplicated regions rather than tandem duplicated clusters. The exon/intron compositions and motif arrangements were considerably conserved among members in the same groups or subgroups. The transcriptional profiles of many duplicated genes were also similar in different soybean tissues even though some of them exhibited divergent expression patterns. The close phylogenetic relationship of *GmLRR-RLKs* and identified *AtLRR-RLK* genes in the same subgroup provided insight into their putative functions. Moreover, some artificial selected *GmLRR-RLKs* have also been identified by comparing the gene diversities of these loci during the evolution from wild to cultivated soybeans. Taken together, all these results provided valuable tools in future efforts to identify specific gene functions of this family and gene diversity among different genotypes of soybean.

## Methods

### *Arabidopsis* LRR-RLKs and soybean genome resources

The amino acid sequences of all *Arabidopsis* LRR-RLKs were acquired from the TAIR database v10.0 (http://www.arabidopsis.org/). The classification of AtLRR-RLKs and nomenclature of groups were based on PlantsP server v.2011 of *Arabidopsis* 2010 project (http://plantsp.genomics.purdue.edu/projects/lrr/Clouse2010.htm) [66]. The genomic, coding and amino-acid sequences of all annotated soybean genes were according to genome sequence of *Glycine max* Wm82.a2.v1 from Phytozome v10 (http://phytozome.jgi.doe.gov/pz/portal.html) [67].

### Identification of *LRR-RLK* genes in soybean genome

The amino-acid sequences of all *Arabidopsis* LRR-RLK members were used to run a local blast search against the protein database of all annotated soybean genes by using Bioedit v7 [68] and all proteins with an E-value less than 10^−6^ were selected as putative soybean LRR-LRKs. These putative GmLRR-RLKs were further filtered by removing redundant sequences and functional annotation, following by analysis with SMART (http://smart.embl-heidelberg.de) [69] and PFAM (http://pfam.xfam.org/) [70] to ensure the presence of LRR and kinase domains.

### Multiple sequence alignments and phylogenetic tree construction

The amino-acid sequence of kinase domain for each GmLRR-RLK and AtLRR-RLK protein was extracted after prediction of kinase domains from these proteins. Multiple sequence alignments were performed by using ClustalX (version 1.83) with default parameters [71]. Unrooted phylogenetic trees were constructed for soybean LRR-RLKs alone or soybean/*Arabidopsis* together with MEGA 6.0 [72] using the neighbor-joining (NJ) method. The nodes were tested by bootstrap analysis with 1000 replicates and the tree with the highest likelihood was selected for further analysis.

### The chromosome location, gene structure, and motif analysis of the soybean *LRR-RLK* genes

All members of *GmLRR-RLKs* were mapped onto soybean chromosomes based on the physical positions of them. The image of chromosomal location was produced with MapInspect software (http://mapinspect.software.informer.com). The number and positions of exons and introns for soybean LRR-RLK genes were determined by comparison of the coding sequences with their corresponding genomic DNA sequences using GSDS v2.0 [73]. The presence of signal peptides and transmembrane domains was predicted with Signalp v4.1 (http://www.cbs.dtu.dk/services/SignalP/) [74], TMHMM v2.0 (http://www.cbs.dtu.dk/services/TMHMM/) [75] and Phobius (http://phobius.sbc.su.se/) [76] respectively. The combination of phylogenetic tree, gene and protein structures was generated using iTOL tool (http://itol.embl.de) [77].

### Duplication analysis and calculating the date of duplication events

Tandem duplications were characterized as multiple members of this gene family occurring within neighboring intergenic regions. In this study, soybean *LRR-RLK* genes clustered together within 200 kb were regard as tandem duplicated genes based on the criteria of other plants in previous reports [28, 78]. The segmental duplicated *GmLRR-RLKs* were characterized according to the PGDD database (http://chibba.agtec.uga.edu/duplication/). The *Ks* and *Ka* values for duplicated gene pairs were also calculated by using PGDD database. The *Ks* values were used to calculate the approximately dates of duplication events and the clock-like rate (λ) of synonymous substitution was set 6.1x10^−9^ substitutions/synonymous site/year for soybean [32, 79, 80].

### Transcriptional profile analysis

RNA-seq data of soybean tissues for roots, root hairs, nodules, leaves, stems, flowers, SAM, pods, and seeds was obtained from Phytozome v10 (http://phytozome.jgi.doe.gov/pz/portal.html) and the expression profiles of all *GmLRR-RLKs* were selected for further analysis. Soybean *LRR-RLK* genes were clustered based on the expression profiles and hierarchical clustering of transcriptional data was performed with MultiExperiment Viewer (Mev) v.4.9.0 using Pearson correlation and Average Linkage Clustering algorithm [81].

### Quantitative real time RT-PCR analysis

Soybean plants (ecotype Williams 82) were grown on soil in the chamber under long day conditions (16 h light/8 h dark cycle) at 25 ± 1 °C. Roots, stems, simple leaves, trifoliolate leaves, shoot apical meristem (SAM) from 2-week-old seedlings, flowers, pods and 1-week-old seedlings were collected for total RNA isolation. Total RNA was extracted using TRIzol Reagent (Invitrogen, USA) and was treated with RNase-free DNase (TaKaRa, Japan). Five micrograms of total RNA were reverse-transcribed using the ReverTra Ace qPCR RT Kit (TOYOBO, Japan) in a reaction of 20 μL. The cDNA was diluted 50 times as the template for quantitative RT-PCR. The PCR amplification was carried out on an Applied Biosystems 7300 Real-Time PCR System, using SYBR Premix Ex Taq kit (TaKaRa, Japan). The procedure of the reaction was set according to the manufacturer’s protocol and sequences of primers used were shown in Additional file 12. The relative expression level of each gene, corresponding to the expression level of Actin, was calculated using 2^−ΔΔt^ method [82].

### Selective analysis of *GmLRR-RLKs* among *soja* genus

SNP data of 25 wild soybeans and 31 cultivated soybeans were downloaded from NCBI web site (http://www.ncbi.nlm.nih.gov/SNP/snp_viewTable.cgi?handle=NFCRI_MOA_CAAS). SNP loci of the *GmLRR-RLKs* were identified based on the physical position of each gene. The v1.1 version of soybean gene annotation was used since the physical positions of all SNPs were based on this version of soybean genome. The gene diversity of each SNP loci in *G.max* and *G.soja*, and *F*_*st*_ value were calculated by Genepop V4.0 [83]. The SNP locus with *F*_*st*_ >0.45 was defined as a putative selective site during domestication.

### Availability of data and materials

The data supporting the results of this article is included within the article and its additional files.

## Additional files

Additional file 1: List of identified *LRR-RLK* genes in soybean. The ID, gene code, gene length, physical position on chromosome, number of exon/intron/UTR, length of amino-acid/signal peptide, number and position of TM, position of kinase domain for each soybean *LRR-RLK* gene annotated in this study were included. (XLSX 66 kb) Additional file 2: Unrooted phylogenetic tree of GmLRR-RLKs and AtLRR-RLKs. The sequences of kinase domains from 467 GmLRR-RLKs and 213 AtLRR-RLKs were aligned by Clustal X 1.8.3 and the phylogenetic tree was constructed using the MEGA 6.0 by the neighbor-joining with 1000 bootstrap replicates. (PDF 1599 kb) Additional file 3: The exon/intron organization of all soybean *LRR-RLK* genes. Exons are represented by yellow boxes and introns by black lines. UTR regions of some genes are also indicated using blue boxes. The relative sizes of exons, introns and UTR can be estimated by the length of boxes or lines. (PDF 86 kb) Additional file 4: Putative motifs of all GmLRR-RLKs in each subgroup predicted by MEME. Motifs were identified by MEME software using the deduced amino-acid sequences of GmLRR-RLKs in each group and the relative position of each identified motif was shown. (XLSX 3460 kb) Additional file 5: The pattern of signal peptides, LRRs, TMs, and kinases for all GmLRR-RLKs. The signal peptide, transmembrane domain, and kinase domain are represented by black, red and blue boxes respectively. LRR motifs are indicated using green oval shapes. The relative size of each motif can be estimated by the length. (PDF 205 kb) Additional file 6: Soybean *LRR-RLK* genes located in tandem duplication clusters. A region containing two or more soybean *LRR-RLK* genes within 200 kb was defined as a tandem duplication cluster. The gene ID, subgroups, and chromosome of each *GmLRR-RLK* located in tandem duplication clusters were presented. (XLSX 11 kb) Additional file 7: Estimates of the dates for the segmental duplication events of *LRR-RLK* gene family in soybean. (XLSX 21 kb) Additional file 8: Expression profiles for all soybean *LRR-RLK* genes across different tissues. The genome-wide RNA-seq data of soybean were obtained from Phytozome v10. The expression data of *GmLRR-RLKs* in pod, root hair, leaves, root, nodules, seed, stem, SAM, flower was gene-wise normalized and hierarchically clustered. The color scale below represents expression values, green indicating low levels while red indicating high levels of transcript abundance. (PDF 7242 kb) Additional file 9: Comparison of expression pattern for selected tandem duplicated gene pairs by qRT-PCR and RNA-seq dataset. The expression levels of two tandem duplicated gene pairs in different organs analyzed by quantitative RT-PCR (A and C) were consistent with the pattern identified from RNA-seq dataset (B and D). The expression level in the root for each gene was set to 1.0, and error bars represented standard errors of three biological replicates. (PDF 140 kb) Additional file 10: SNP loci located in soybean *LRR-RLK* genes identified by analysis of the resequencing data of 25 wild soybeans and 31 cultivated soybeans. (XLSX 2377 kb) Additional file 11: Putative artificial selected *GmLRR-RLKs* during soybean domestication. (XLSX 15 kb) Additional file 12: The primers used for quantitative real time RT-PCR. (PDF 6 kb)
