# Supplementary figures and images for: Genome-wide identification and evolutionary analysis of leucine-rich repeat receptor-like protein kinase genes in soybean
Source: BMC Plant Biol. 2016 Mar 2;16:58. doi: 10.1186/s12870-016-0744-1 (PMC4776374; doi:10.1186/s12870-016-0744-1)

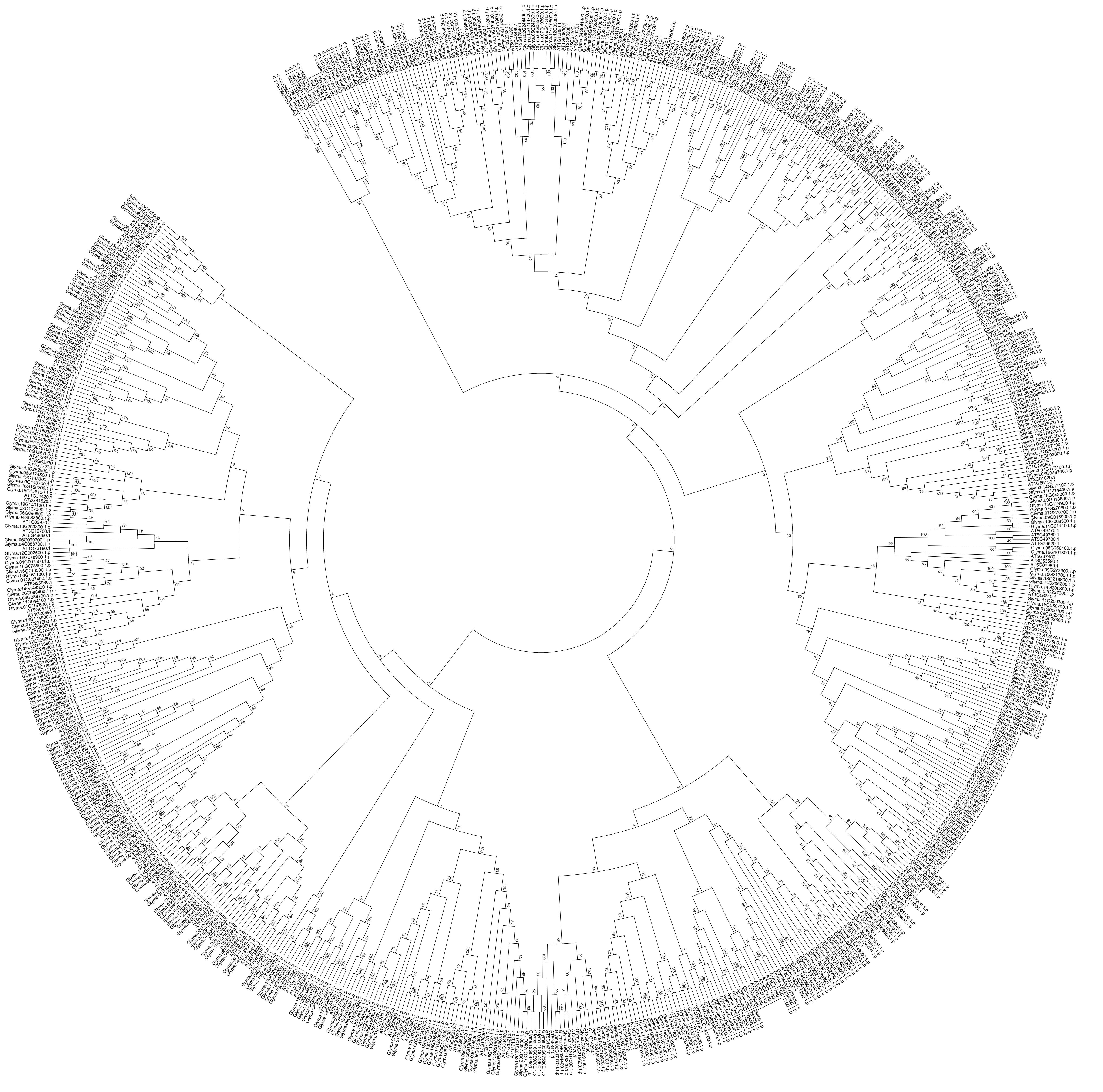

Supplement: Additional file 2: — Unrooted phylogenetic tree of GmLRR-RLKs and AtLRR-RLKs. The sequences of kinase domains from 467 GmLRR-RLKs and 213 AtLRR-RLKs were aligned by Clustal X 1.8.3 and the phylogenetic tree was constructed using the MEGA 6.0 by the neighbor-joining with 1000 bootstrap replicates. (PDF 1599 kb) [file 12870_2016_744_MOESM2_ESM.pdf]

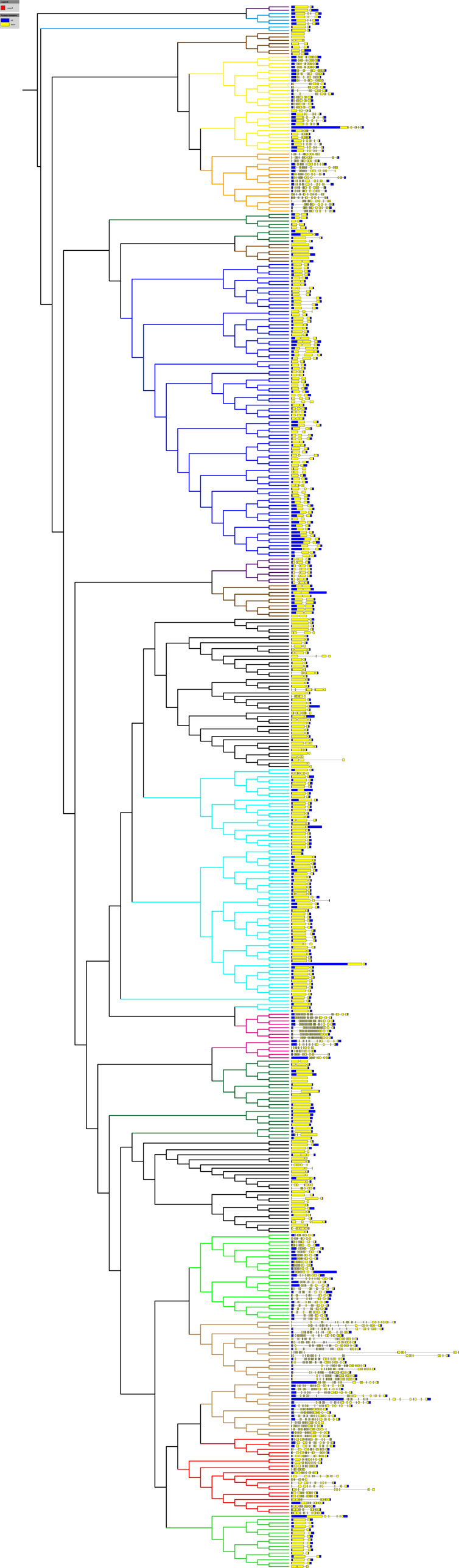

Supplement: Additional file 3: — The exon/intron organization of all soybean LRR-RLK genes. Exons are represented by yellow boxes and introns by black lines. UTR regions of some genes are also indicated using blue boxes. The relative sizes of exons, introns and UTR can be estimated by the length of boxes or lines. (PDF 86 kb) [file 12870_2016_744_MOESM3_ESM.pdf]

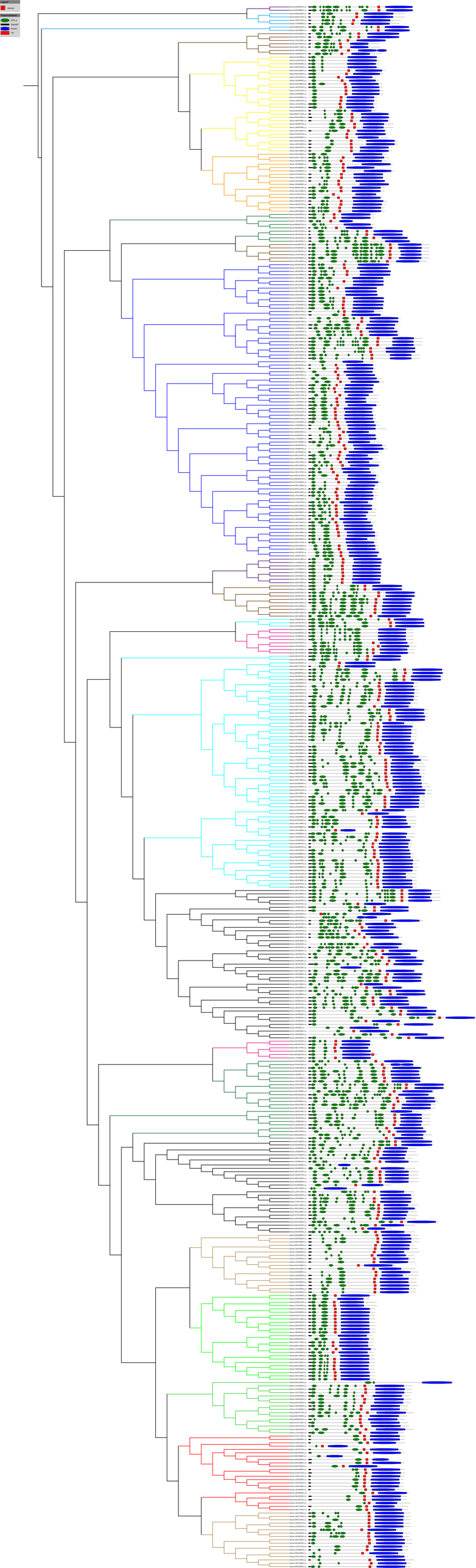

Supplement: Additional file 5: — The pattern of signal peptides, LRRs, TMs, and kinases for all GmLRR-RLKs. The signal peptide, transmembrane domain, and kinase domain are represented by black, red and blue boxes respectively. LRR motifs are indicated using green oval shapes. The relative size of each motif can be estimated by the length. (PDF 205 kb) [file 12870_2016_744_MOESM5_ESM.pdf]

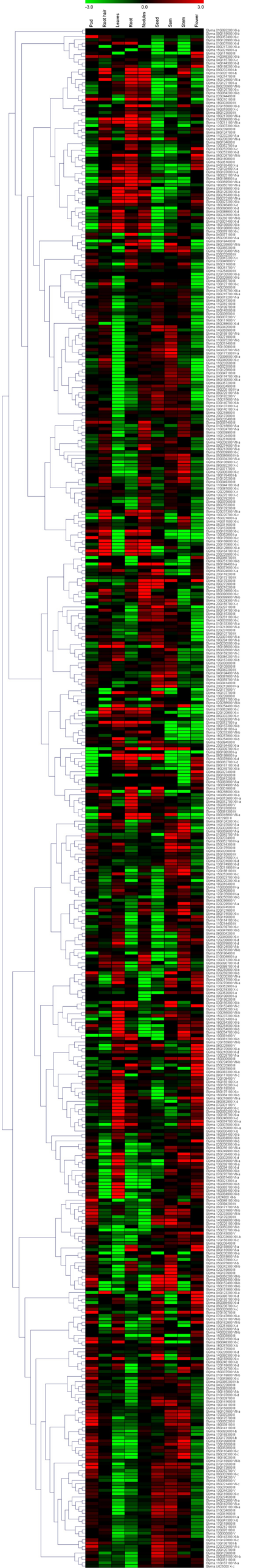

Supplement: Additional file 8: — Expression profiles for all soybean LRR-RLK genes across different tissues. The genome-wide RNA-seq data of soybean were obtained from Phytozome v10. The expression data of GmLRR-RLKs in pod, root hair, leaves, root, nodules, seed, stem, SAM, flower was gene-wise normalized and hierarchically clustered. The color scale below represents expression values, green indicating low levels while red indicating high levels of transcript abundance. (PDF 7242 kb) [file 12870_2016_744_MOESM8_ESM.pdf]

**A****09g018800,09g018900 qRT-PCR**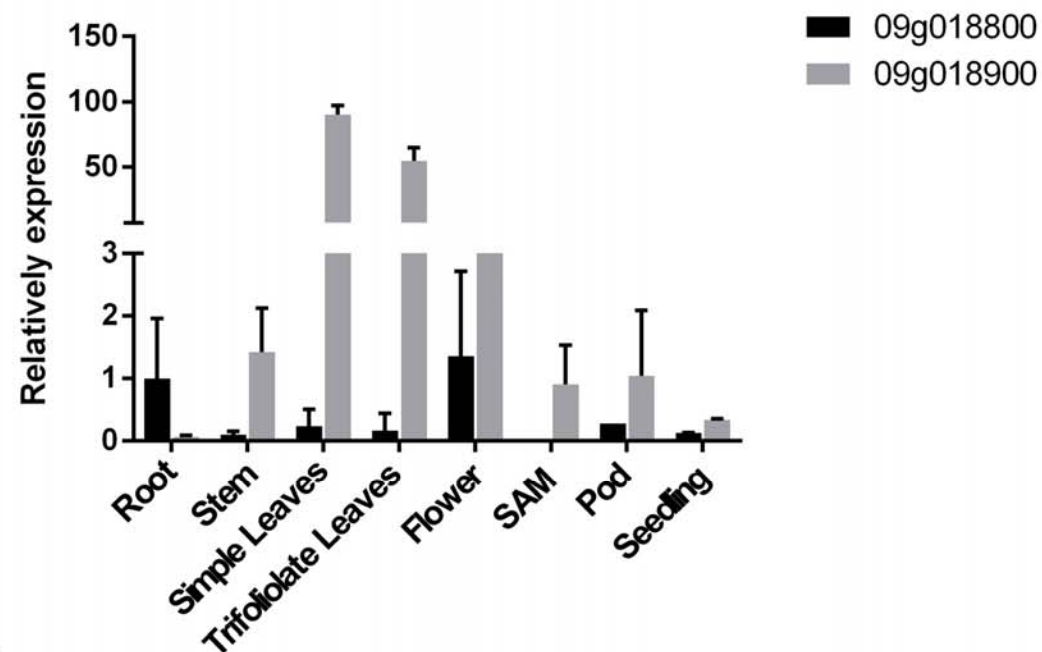**B****09g018800,09g018900 Phytozome**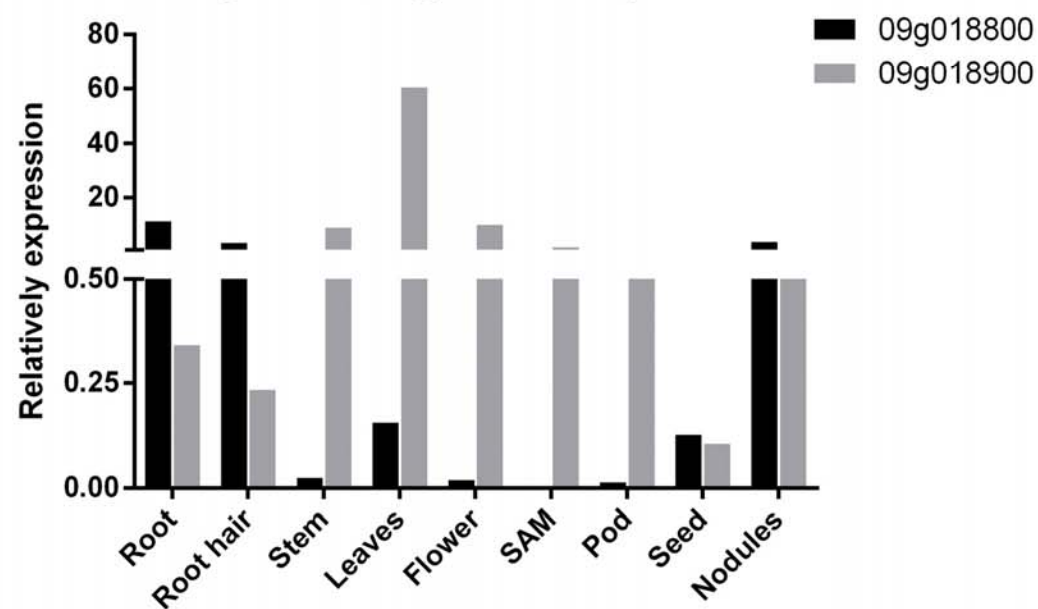**C****16g156100,16g156200 qRT-PCR**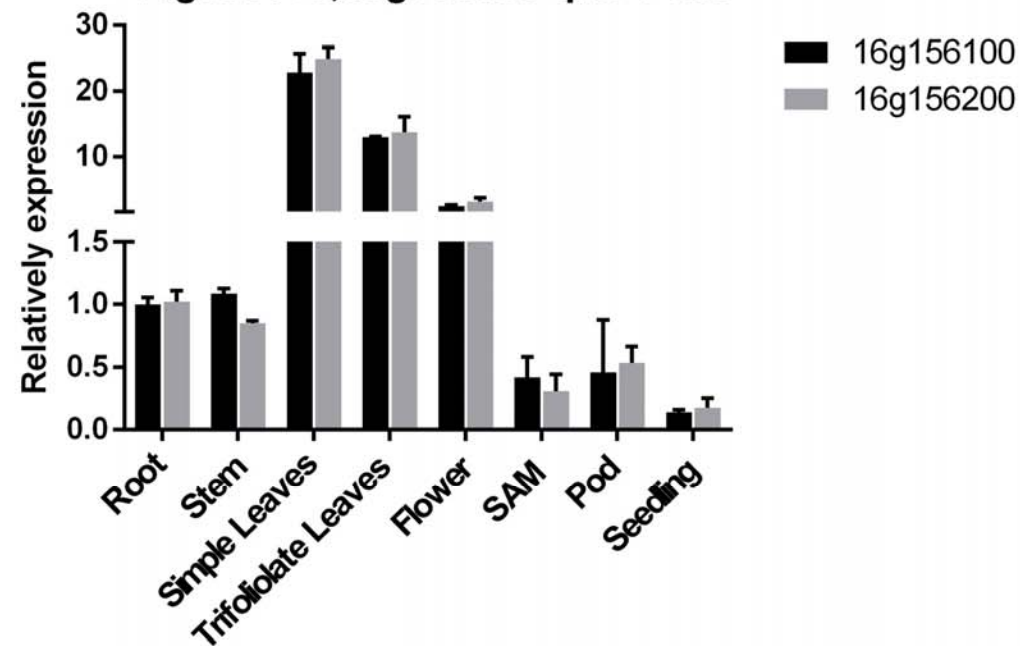**D****16g156100,16g156200 Phytozome**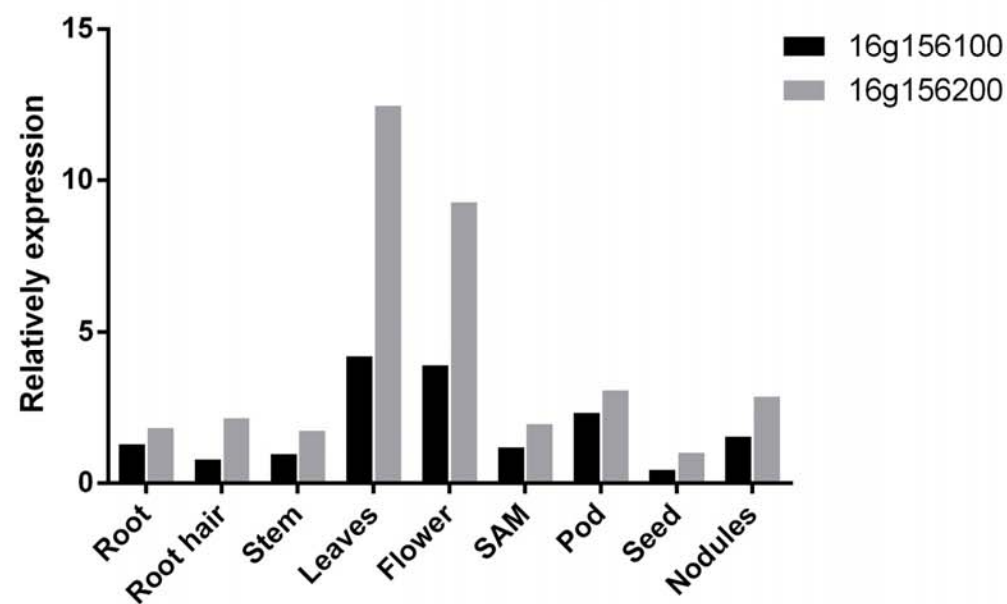

Supplement: Additional file 9: — Comparison of expression pattern for selected tandem duplicated gene pairs by qRT-PCR and RNA-seq dataset. The expression levels of two tandem duplicated gene pairs in different organs analyzed by quantitative RT-PCR (A and C) were consistent with the pattern identified from RNA-seq dataset (B and D). The expression level in the root for each gene was set to 1.0, and error bars represented standard errors of three biological replicates. (PDF 140 kb) [file 12870_2016_744_MOESM9_ESM.pdf]
