# Supplementary material for: Genome-wide identification and evolutionary analysis of leucine-rich repeat receptor-like protein kinase genes in soybean
Source: BMC Plant Biol. 2016 Mar 2;16:58. doi: 10.1186/s12870-016-0744-1 (PMC4776374; doi:10.1186/s12870-016-0744-1)
Supplement: Additional file 12: — The primers used for quantitative real time RT-PCR. (PDF 6 kb) [file 12870_2016_744_MOESM12_ESM.pdf]

Primers used for quantitative real time RT-PCR:

Glyma.09G018800
Forward: 5' GCCAATAGAGCGAGGGAAATA 3'
Reverse: 5' TTCTCAAAACCGCTCAGTGC 3'

Glyma.09G018900
Forward: 5' CTGGAGGGTTTTGAGAAGTTTGT 3'
Reverse: 5' GTTACTGATTCAGATGTTAGGTGCA 3'

Glyma.16G156100
Forward: 5' CTTCATGGAAGCATGTTAGATTGG 3'
Reverse: 5' GTGTATGCATACTCTGGAGGA 3'

Glyma.16G156200
Forward: 5' AAACTCTTTAGATTGGGCTAGCAG 3'
Reverse: 5' TGTGTATGCATACTCTGGAGG 3'

Actin
Forward: 5' CTTCCCTCAGCACCTTCCAA 3'
Reverse: 5' GGTCCAGCTTTCACACTCCAT 3'
